# Cutaneous Sensory Stimulation Intensity Modulates Beta‐Band Event‐Related Desynchronization and Synchronization Amplitudes

**DOI:** 10.1111/ejn.70613

**Published:** 2026-07-11

**Authors:** Rin Kosuge, Mayu Akaiwa, Hidekazu Saito, Yuya Matsuda, Koki Iwata, Eriko Shibata, Takeshi Sasaki, Jun Shinozaki, Masanori Sasaki, Yuki Ueda, Kazuhiro Sugawara

**Affiliations:** ^1^ Graduate School of Health Sciences Sapporo Medical University Sapporo Hokkaido Japan; ^2^ Department of Human Health Sciences, Graduate School of Medicine Kyoto University Kyoto Japan; ^3^ Department of Physical Therapy, School of Health Sciences Sapporo Medical University Sapporo Hokkaido Japan; ^4^ Department of Occupational Therapy, School of Health Sciences Sapporo Medical University Sapporo Hokkaido Japan; ^5^ Department of Rehabilitation Sapporo Hakuyokai Hospital Sapporo Hokkaido Japan; ^6^ Department of Rehabilitation, Faculty of Health Sciences Hokkaido Bunkyo University Eniwa Hokkaido Japan; ^7^ Division of Neuroscience, Department of Physiology, School of Medicine, Sapporo Medical University Sapporo Medical University Sapporo Hokkaido Japan; ^8^ Department of Pediatrics Hokkaido University Hospital Sapporo Hokkaido Japan

**Keywords:** beta‐band oscillations, cutaneous stimulation, magnetoencephalography, stimulus intensity

## Abstract

Beta‐band event‐related desynchronization and synchronization elicited by somatosensory stimulation could be potential neurophysiological markers of motor function, specifically in neurological disorders; however, their characteristics in healthy individuals remain unclear. This study aimed to examine whether the amount of afferent input modulates beta‐band oscillatory responses and if they are associated with motor performance in healthy adults. Magnetoencephalographic data were recorded for 24 healthy participants during cutaneous electrical stimulation of the right index finger at three intensities based on individual sensory threshold (1×, 2× and 3× thresholds). Amplitudes of beta‐band event‐related desynchronization and synchronization were quantified relative to a prestimulus baseline, and motor performance was assessed using the Assembly test of the Purdue Pegboard Test. Neither oscillatory response showed a significant association with motor performance. However, the proportion of participants exhibiting these responses increased with increasing stimulation intensity, indicating reduced interindividual variability at higher intensities. Amplitudes of both responses increased with higher afferent input but showed limited sensitivity to subtle differences in input amount. These results demonstrate that cutaneous stimulation can elicit beta‐band oscillations in healthy individuals, reflecting afferent input and being possibly modulated by its amount. These findings contribute to improved understanding of beta‐band oscillatory responses, specifically to afferent input magnitude.

## Graphical abstract

Beta‐band oscillations (βERD and βERS) were examined under different levels of cutaneous electrical stimulation. Both the amplitude and occurrence of βERD and βERS increased with stimulus intensity, whereas neither βERD nor βERS power was correlated with motor performance in healthy individuals. These findings contribute to a physiological understanding of beta‐band oscillatory responses, particularly in relation to afferent input.

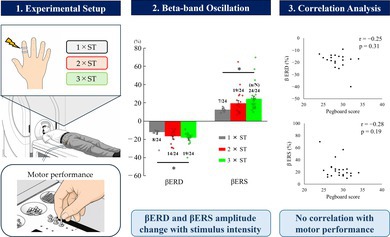

**Abbreviations**
βERD: beta event‐related desynchronization
βERS: beta event‐related synchronization
EOG: electrooculogram
GABA: gamma‐aminobutyric acid
HPI: head position indicator
LMMs: linear mixed models
M1: primary motor cortex
MEG: magnetoencephalography
RMS: root mean square
S1: primary somatosensory cortex
SEF: somatosensory evoked magnetic field
ST: sensory threshold
TFA: time–frequency analysis
TSE: temporal spectral evolution

## 1 Introduction

Voluntary movements are associated with modulation of beta‐band (13–30 Hz) oscillations in the contralateral sensorimotor area (Pfurtscheller and Lopes da Silva 1999; Cassim et al. 2001; Houdayer et al. 2006; Gaetz et al. 2011). A decrease in beta‐band power relative to rest, known as beta event‐related desynchronization (βERD), begins just before movement onset and continues during execution. This is typically followed by a post‐movement increase in power, referred to as beta event‐related synchronization (βERS). βERD and βERS occur not only during voluntary movement but also in response to somatosensory stimulation (Houdayer et al. 2006; Parkkonen et al. 2018; Illman et al. 2020) and following passive movements (Cassim et al. 2001; Parkkonen et al. 2017, 2018; Illman et al. 2020). These findings suggest that afferent input related to voluntary movement plays a critical role in generating βERD and βERS. Cassim et al. (2001) reported that βERS is abolished during passive movement when afferent input is blocked via ischaemia.

The relationship between afferent input and βERS has been extensively investigated (Houdayer et al. 2006; Parkkonen et al. 2017, 2018; Illman et al. 2020). Houdayer et al. (2006) compared cutaneous, single and repeated stimulation of the median nerve, concluding that βERS is modulated based on sensory stimulation type. However, the study lacked a quantitative assessment of afferent input, making it unclear how changes in input strength quantitatively affect βERS. A previous study that controlled for muscle contraction strength found that βERS amplitude increases with stronger contractions, whereas βERD amplitude remains largely unaffected (Fry et al. 2016). Stronger muscle contractions increase the firing rates of muscle spindles and tendon organs (Jami 1992; Boulton et al. 2021) and likely enhance skin receptor activation due to increased pressure at contact site. Thus, the greater βERS amplitude observed during stronger contractions may reflect increased afferent input. Furthermore, previous studies on beta‐band oscillatory modulations primarily focused on amplitude changes (Stancak et al. 2003; Illman et al. 2020; Houdayer et al. 2006). However, some individuals do not exhibit detectable βERD or βERS after sensory stimulation (Houdayer et al. 2006; Stancak et al. 2003), suggesting that amplitude modulations alone may not fully capture the response characteristics of βERD and βERS to the afferent input magnitude. Thus, the present study aimed to investigate not only whether afferent input magnitude modulates the amplitude of beta‐band oscillations but also how the occurrence rate of these responses changes with input level variations.

This study aimed to determine whether the magnitude of afferent input modulates β‐band oscillations. Common sensory stimuli used to evoke βERD and βERS include median nerve stimulation, tactile stimulation and passive movement (Houdayer et al. 2006; Parkkonen et al. 2017; Illman et al. 2020). However, these approaches activate cutaneous and proprioceptive pathways, making it difficult to quantify total afferent input. To address this, we used electrical stimulation of the skin to isolate cutaneous sensation from other somatosensory inputs and to quantify the level of stimulation required to induce βERD and βERS, based on sensory thresholds (STs). Furthermore, somatosensory evoked field (SEF) analysis was performed to verify the afferent input magnitude reaching the cortex under each stimulation intensity condition, ensuring that our experimental manipulation of stimulation intensity was reflected at the cortical level.

Changes in β‐band oscillations have been linked to the severity and recovery of motor function in patients with neurological disorders (Peter et al. 2022). In stroke patients, βERD and βERS elicited by voluntary movement or sensory stimulation on the affected side have been correlated with motor performance (Parkkonen et al. 2018; Tang et al. 2020). Moreover, in the acute phase of stroke, βERS has been associated with motor outcomes at 1 and 12 months post‐stroke (Parkkonen et al. 2017, 2018), suggesting its potential as a biomarker for motor recovery. However, few studies have explored the relationship between βERD/βERS and motor performance in healthy individuals; therefore, whether these associations are observable in healthy populations or reflect alterations in cortical states associated with disease remains unclear.

This study had three main objectives: (1) to examine whether the magnitude of afferent input modulates βERD and βERS, (2) to determine whether βERD and βERS are associated with motor performance and (3) to investigate whether the occurrence rates of βERD and βERS are modulated by the afferent input magnitude. By testing the need for considering not only amplitude modulations but presence/absence of responses and differences in study populations when interpreting βERD and βERS, this study provides significant insights into the neural foundations of beta‐band oscillatory changes.

## 2 Materials and Methods

### 2.1 Participants

The minimum required sample size for this study was estimated using G*Power software version 3.1.9.7 (Heinrich‐Heine‐Universität Düsseldorf, Düsseldorf, Germany) based on the partial eta‐squared value (η^2^p = 0.067) for differences in βERS intensity across within‐subject conditions, as previously reported (Tatti et al. 2019). The chosen effect size of 0.27, with a statistical power of 0.80 and an alpha level of 0.05, resulted in a minimum sample size of 24 participants. Accordingly, we recruited 24 healthy young adults [age (mean ± standard deviation [SD]) 24.4 ± 2.2 years; 14 men and 10 women] without a history of neurological disorders. All participants received a detailed explanation of the experimental procedures before participation and provided written informed consent. This study was approved by the ethics committee of Sapporo Medical University (approval number: 6–1‐26) and conducted following the tenets of the Helsinki Declaration (1964) and later amendments.

### 2.2 Performance Test

Motor performance was assessed using the Assembly test of the Purdue Pegboard Test (Tiffin and Asher 1948), an international standardized measure of fine motor performance. In this test, participants assemble a unit by sequentially (1) inserting a pin into a hole, (2) placing a washer onto the pin, (3) adding a collar and (4) placing a second washer on top of the collar. The performance score was the number of completed assemblies within 1 min. Although the standard Assembly test is typically performed using both hands, only the right hand was used here as in Matsuura et al. (2020).

### 2.3 Electrical Stimulation

Participants wore ring electrodes on the right index finger for somatosensory stimulation; electrical stimuli were delivered using electrical stimulation equipment (SEN; Nihon Kohden, Tokyo, Japan). The stimulus duration was set to 0.2 ms. Before the experiment, we determined each participant's ST, defined as the minimum stimulus intensity at which the electrical stimulation was perceived. We then delivered electrical stimulation at three intensity levels relative to ST: 1 × ST (1 × ST condition), 2 × ST (2 × ST condition) and 3 × ST (3 × ST condition). At all intensity levels, no visible muscle contractions were observed during stimulation. A previous study showed that when the index finger is electrically stimulated at 3 × ST, βERS returns to baseline ~3000 ms after stimulus onset (Houdayer et al. 2006). Therefore, to avoid carryover effects from the preceding stimulus, the interstimulus interval was randomized between 4000 and 6000 ms. A total of 300 stimuli were delivered for each condition, consisting of two sets of 150 stimuli. During electrical stimulation, participants were comfortably positioned in a supine position, with the right upper limb placed alongside the body and the forearm in a pronated position. In addition, participants were instructed to fixate on a visual fixation point on the ceiling in front of them and to remain as still as possible.

### 2.4 Magnetoencephalography Recordings

Magnetoencephalography signals were recorded using a whole‐head 306‐channel MEG system (Neuromag; Elekta, Helsinki, Finland) in a magnetically shielded room. To reject trials with eye blinks, an electrooculogram (EOG) was recorded from a pair of electrodes placed on the supra‐ and infra‐orbit of the right eye using a Neurofax system (Nihon Kohden, Tokyo, Japan). The recording passband was 0.10–200 Hz for MEG signals and 0.53–120 Hz for EOG signals. The sampling rate was 600 Hz for MEG and 1000 Hz for EOG. Before MEG recording, four head position indicator (HPI) coils were attached to the skin in the forehead and behind the ears. The locations of HPI coils and three anatomical fiducial points (the nasion and left and right preauricular points) were digitized using a three‐dimensional digitizer (Hamalainen et al. 1993; Saito et al. 2021).

### 2.5 Data Analysis

#### 2.5.1 Preprocessing

MEG and EOG data were analysed using MATLAB R2019a (MathWorks, Natick, MA, USA). First, external noise originating from inside and outside the sensor array was excluded using the temporal extension of the signal space separation method implemented in MaxFilter software (version 2.2; Elekta Oy, Helsinki, Finland) (Taulu and Kajola 2005). Then, data were segmented into epochs ranging from 1000 ms before to 3000 ms after stimulus onset. Epochs with a 50‐Hz notch‐filtered EOG signal of > 150 μV were excluded from further analysis (Nishitani and Hari 2000; Jones et al. 2007).

#### 2.5.2 Analysis of Somatosensory Evoked Magnetic Fields

SEF analysis was performed using signals from 204‐channel planar‐type gradiometers. For each condition, epochs were averaged, and the root mean square (RMS) of the magnetic field signals was calculated from each pair of gradiometers over the left sensorimotor area. The mean of prestimulus samples from −500 to −100 ms was used as baseline. Based on the RMS waveform in the 3 × ST condition, peak channels showing the greatest amplitude were identified for the M25, M40 and M60 components, typically appearing at ~25, 40 and 60 ms after stimulus onset, respectively (Kida et al. 2018). Finally, using the peak channels identified in the 3 × ST condition, the latencies and amplitudes of the M25, M40 and M60 components were quantified for all conditions.

#### 2.5.3 Analysis of β‐Band Oscillations

Signals from 204‐channel planar‐type gradiometers were used to analyse β‐band oscillations, as in SEF analysis. To visualize stimulus‐induced changes in oscillatory activity, a time–frequency analysis (TFA) was performed. We used Morlet wavelet analysis, as implemented in Brainstorm, based on a mother wavelet with a central frequency of 1 Hz and a temporal resolution of 3 s full width at half maximum (Yaple et al. 2018; Diers et al. 2020). The mean signal in the −500 to −100 ms prestimulus window was used as baseline. βERD and βERS were quantified using the temporal spectral evolution (TSE) method (Salmelin and Hari 1994). Previous studies conducted TSE using a single band‐pass filter across all participants (Enatsu et al. 2014; Parkkonen et al. 2015). However, the peak βERD and βERS frequencies reportedly vary across individuals (Illman et al. 2020; Akaiwa et al. 2024). Therefore, in the present study, TSE was computed for each participant at three frequency bands (13–23, 15–25 and 17–27 Hz); for suppression (βERD) and enhancement (βERS), the frequency band showing the strongest modulation was selected. The baseline was defined as the −500 to −100 ms prestimulus window. βERD and βERS amplitudes were calculated as relative changes (%) to baseline using the formulas $\left(x-\mu \right)/\mu \times 100$ and $\left(y-\mu \right)/\mu \times 100$, respectively. Here, x represents the peak value in the suppressive direction within 0–500 ms after stimulus onset, y represents the peak value in the enhancing direction within 500–1000 ms after stimulus onset and μ the mean baseline value (Parkkonen et al. 2015; Akaiwa et al. 2024). For each participant, peak channels over the left sensorimotor area were identified separately for βERD and βERS based on the strongest modulation in the 3 × ST condition. Using the respective peak channels, βERD and βERS amplitudes were quantified for all stimulation conditions and used for statistical analysis. As previously reported (Enatsu et al. 2014), βERD and βERS were defined as suppression and enhancement of β‐band power exceeding two SDs from the baseline mean, respectively. In the present study, these modulations had to persist for ≥ 100 ms, and the presence or absence of βERD and βERS was determined accordingly. The βERS offset time was defined as the timepoint when the TSE waveform fell below the baseline mean plus two SDs. Furthermore, we conducted a supplementary analysis of αERD and αERS. TFA and TSE analyses were performed using the same method as the beta‐band analysis, with the baseline defined as 500–1000 ms before stimulus onset. The frequency band for TSE analysis was fixed at 8–12 Hz for all participants (Hirata et al. 2002), and amplitudes were calculated as a percentage change relative to the baseline. The time windows for determining αERD and αERS amplitudes differed from those used for the beta‐band; specifically, we identified peak values in the suppressive direction within 0–600 ms and the enhancement direction within 600–1500 ms post‐stimulus, respectively (Della Penna et al. 2004). Similar to beta‐band analysis, occurrence rates and offset times for αERD and αERS were calculated as well.

### 2.6 Statistical Analysis

Statistical analyses were performed using IBM SPSS Statistics version 24 (IBM Corp., Armonk, NY, USA). To assess the effect of stimulus intensity on SEF and the amplitudes of each oscillatory index (βERD, βERS, αERD, αERS), linear mixed models (LMMs) were used, as some participants did not exhibit SEF or ERD/ERS in α‐ or β‐bands. Participants were treated as random effects, and stimulus intensity was included as a fixed effect. When a significant main effect was found, post hoc comparisons were conducted using Bonferroni correction. Normality was assessed for pegboard scores and βERD and βERS amplitudes in the 3 × ST condition. To examine associations between motor performance and βERD/βERS amplitudes, nonparametric Spearman's correlation analyses were conducted between pegboard scores and βERD/βERS amplitudes. A *p*‐value of < 0.05 was considered statistically significant.

## 3 Results

### 3.1 βERD and βERS

**FIGURE 1:**
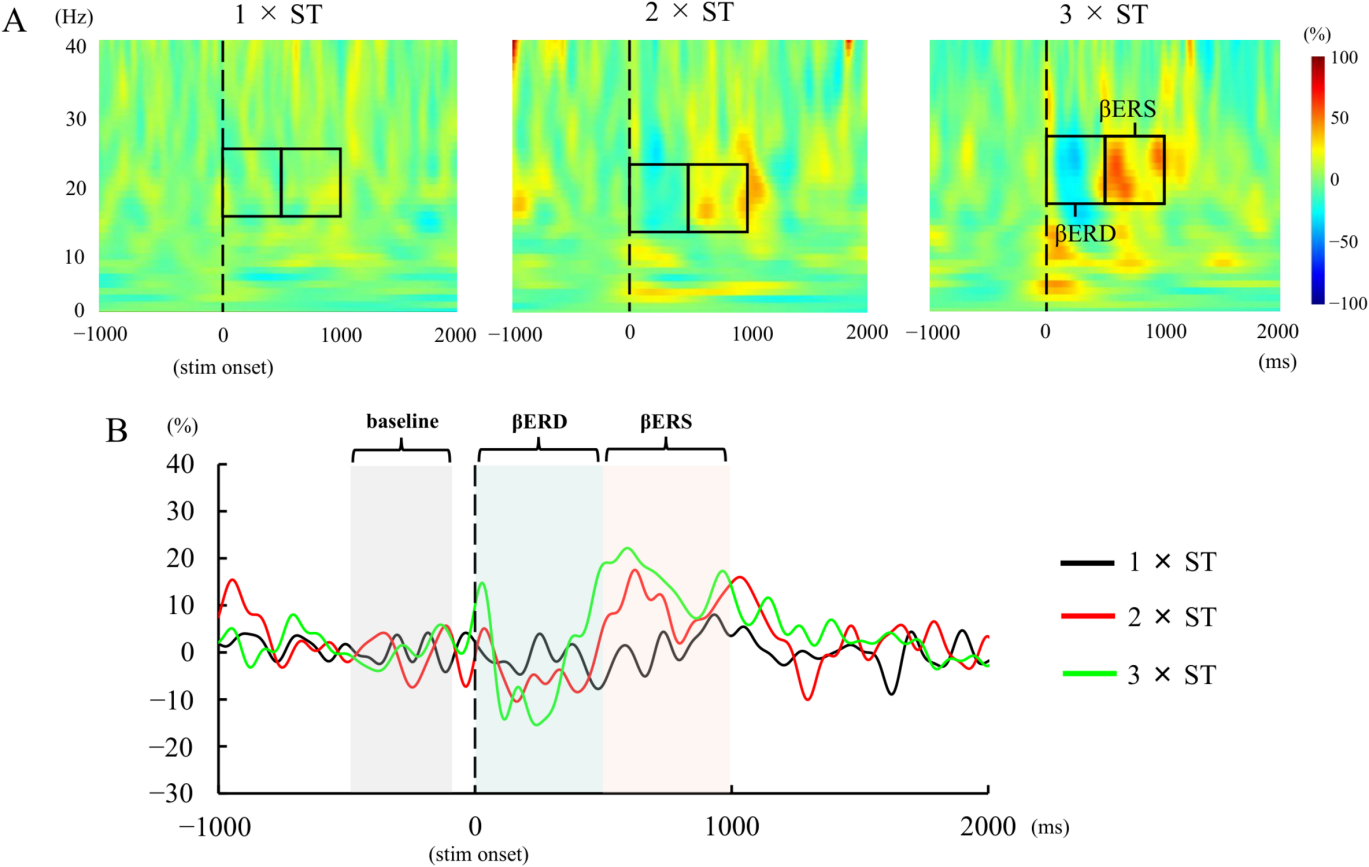
Representative (A) time–frequency analysis (TFA) and (B) temporal spectral evolution (TSE) waveforms for a single participant (sub03). TFA panels (from left to right) correspond to stimulation at 1×, 2× and 3 × the sensory threshold (ST). The two rectangles in each TFA panel indicate the frequency bands and time windows used for the TSE analysis of beta event‐related desynchronization (βERD) and beta event‐related synchronization (βERS), respectively. In TSE waveforms, black, red and green traces represent the 1 × ST, 2 × ST and 3 × ST conditions, respectively. The grey‐shaded area indicates the baseline period used for analysis, and blue‐ and orange‐shaded areas denote the analysis windows for βERD and βERS, respectively.

Table 1 summarizes the βERD and βERS amplitudes for all participants, Figure 1 shows TFA and TSE waveforms for a representative participant. Visual inspection of individual TSE waveforms for all participants confirmed that signals were stable during the baseline period, with no evident trends. Furthermore, since the maximum βERS offset time was 2510 ms post‐stimulus (Supplementary Table S1), it is unlikely that the baseline period was affected by βERS induced by the preceding stimulus. βERD was observed in 8 of 24 participants in the 1 × ST condition, 14 in the 2 × ST condition and 19 in the 3 × ST condition. βERS was observed in 7 of 24 participants in the 1 × ST condition, 19 in the 2 × ST condition and all 24 in the 3 × ST condition.

**TABLE 1:** βERD and βERS amplitudes.

	βERD amplitude (%)			βERS amplitude (%)		
Sub	1 × ST	2 × ST	3 × ST	1 × ST	2 × ST	3 × ST
01	ー	ー	−16	ー	19	23
02	−11	−16	−18	ー	28	29
03	ー	−11	−20	ー	18	22
04	ー	ー	ー	ー	ー	25
05	−10	−25	−18	ー	ー	12
06	ー	−15	−14	16	ー	28
07	ー	ー	ー	9	12	16
08	−11	−17	−15	13	23	24
09	ー	−9	−13	10	29	70
10	−32	−29	−40	ー	21	18
11	ー	−9	−15	12	65	57
12	ー	−16	−16	ー	12	14
13	ー	ー	−17	ー	17	19
14	−13	ー	−18	ー	8	24
15	ー	ー	−9	ー	10	16
16	−13	−13	−19	ー	9	25
17	ー	ー	ー	ー	7	10
18	−13	−29	−25	ー	10	19
19	ー	ー	ー	12	10	15
20	−11	−12	−17	ー	ー	14
21	ー	−30	−22	ー	16	14
22	ー	−20	−24	ー	40	45
23	ー	ー	ー	ー	ー	19
24	ー	ー	−14	15	28	25
Median [IQR]	−12 [−11 to −13]	−16 [−12 to −24]	−17 [−15 to −20]	12 [11–14]	17 [10–25]	21 [15–25]

*Note:* ‘ー’ indicates that βERD/βERS was not observed.

**FIGURE 2:**
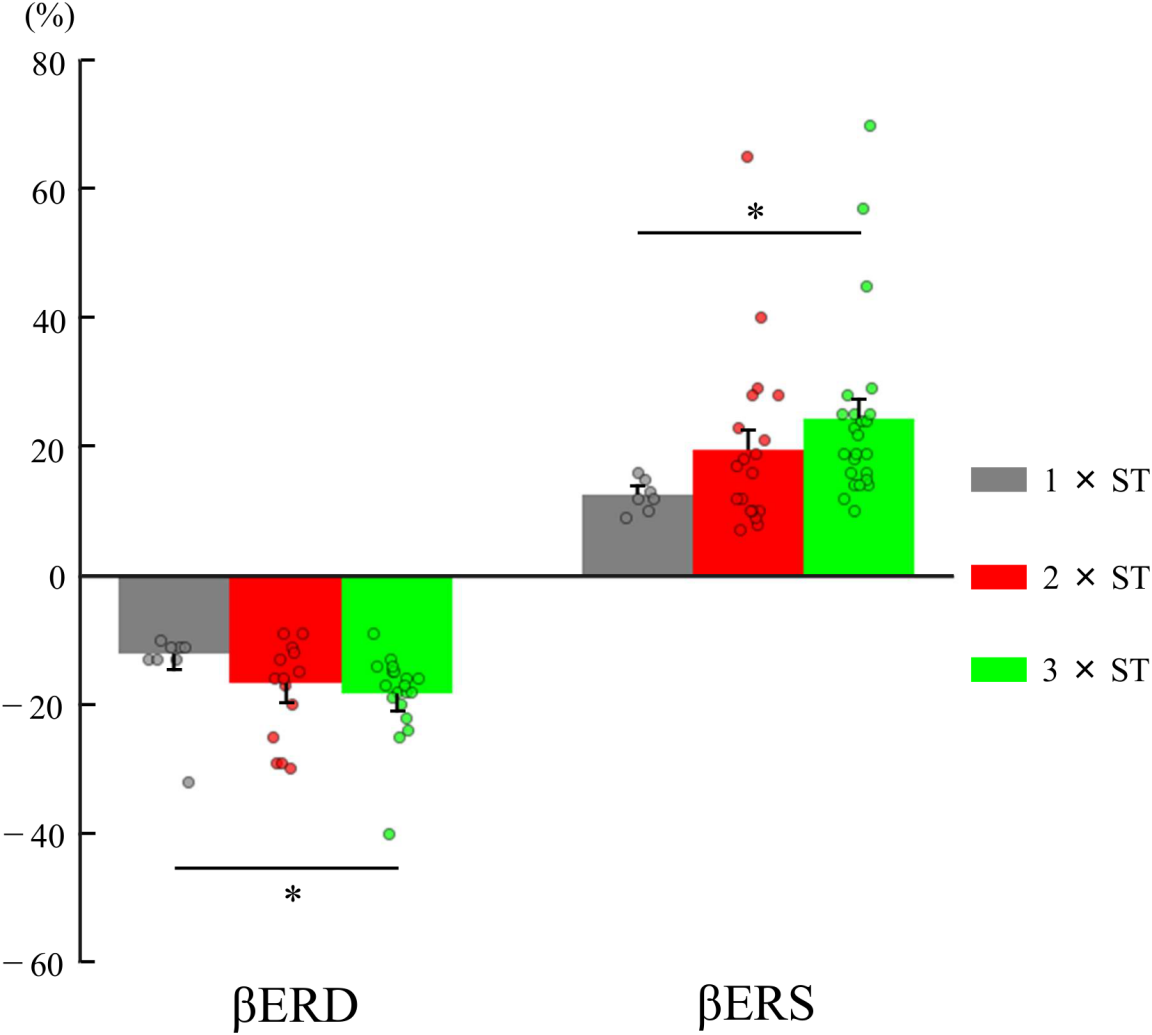
Estimated marginal mean beta event‐related desynchronization (βERD) and beta event‐related synchronization (βERS) amplitudes obtained from the linear mixed model. Error bars indicate the SE. Black squares represent stimulation at 1 × the sensory threshold (1 × ST), red squares 2 × the sensory threshold (2 × ST) and green squares 3 × the sensory threshold (3 × ST); *p < 0.01. Each dot represents an individual participant.

LMMs revealed significant main effects of stimulus intensity on βERD and βERS amplitudes (βERD, *F*_2, 12_ = 49.24, *p* < 0.01; βERS, *F*_2, 22_ = 7.44, *p* < 0.01). Post hoc comparisons with Bonferroni correction showed significantly higher amplitudes in the 3 × ST condition than in the 1 × ST condition for βERD and βERS (βERD, *p* < 0.01; βERS, *p* < 0.01). No significant differences were observed between 1 × ST and 2 × ST conditions (βERD, *p* = 0.07; βERS, *p* = 0.08) or between 2 × ST and 3 × ST conditions (βERD, *p* = 0.86; βERS, *p* = 0.15). Figure 2 shows the estimated marginal means derived from the LMMs.

### 3.2 αERD and αERS

αERD and αERS were subjects of a supplementary analysis. αERD was observed in 9 of 24 participants in the 1 × ST condition, 18 in the 2 × ST condition and 21 in the 3 × ST condition. αERS was observed in 15 of 24 participants in the 1 × ST condition, 14 in the 2 × ST condition and 24 in the 3 × ST condition. A linear mixed model revealed significant main effects of stimulation intensity for both αERD and αERS (αERD, F(2, 13) = 4.16, *p* = 0.04; αERS, F(2, 29) = 9.48, *p* < 0.01). Post hoc tests indicated that αERS was significantly greater in the 3 × ST condition than in the 1 × ST condition (*p* < 0.01). No significant differences were found between any other pairs of conditions; for αERD, no significant differences were observed between any conditions. Representative data (Figure S1), offset times (Table S1), individual amplitude values for all participants (Table S2), and LMM results (Figure S2) are provided in the Supporting Information.

### 3.3 SEF Amplitude

**FIGURE 3:**
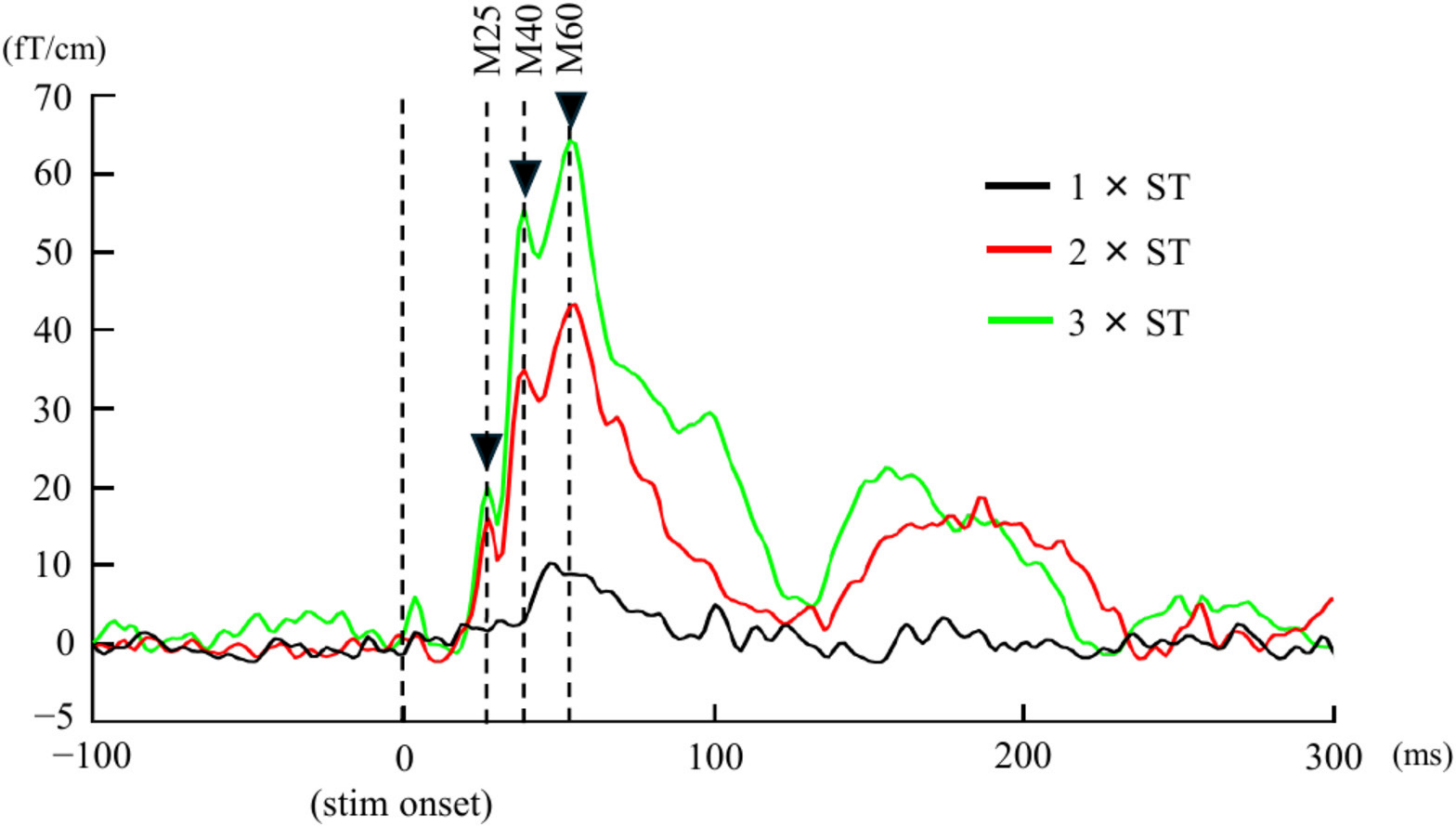
Representative SEF waveforms for a single participant (sub03). Black, red and green traces represent stimulation at 1×, 2× and 3 × the sensory threshold (ST), respectively. Black arrows indicate the M25, M40 and M60 components in the 3 × ST condition.

**FIGURE 4:**
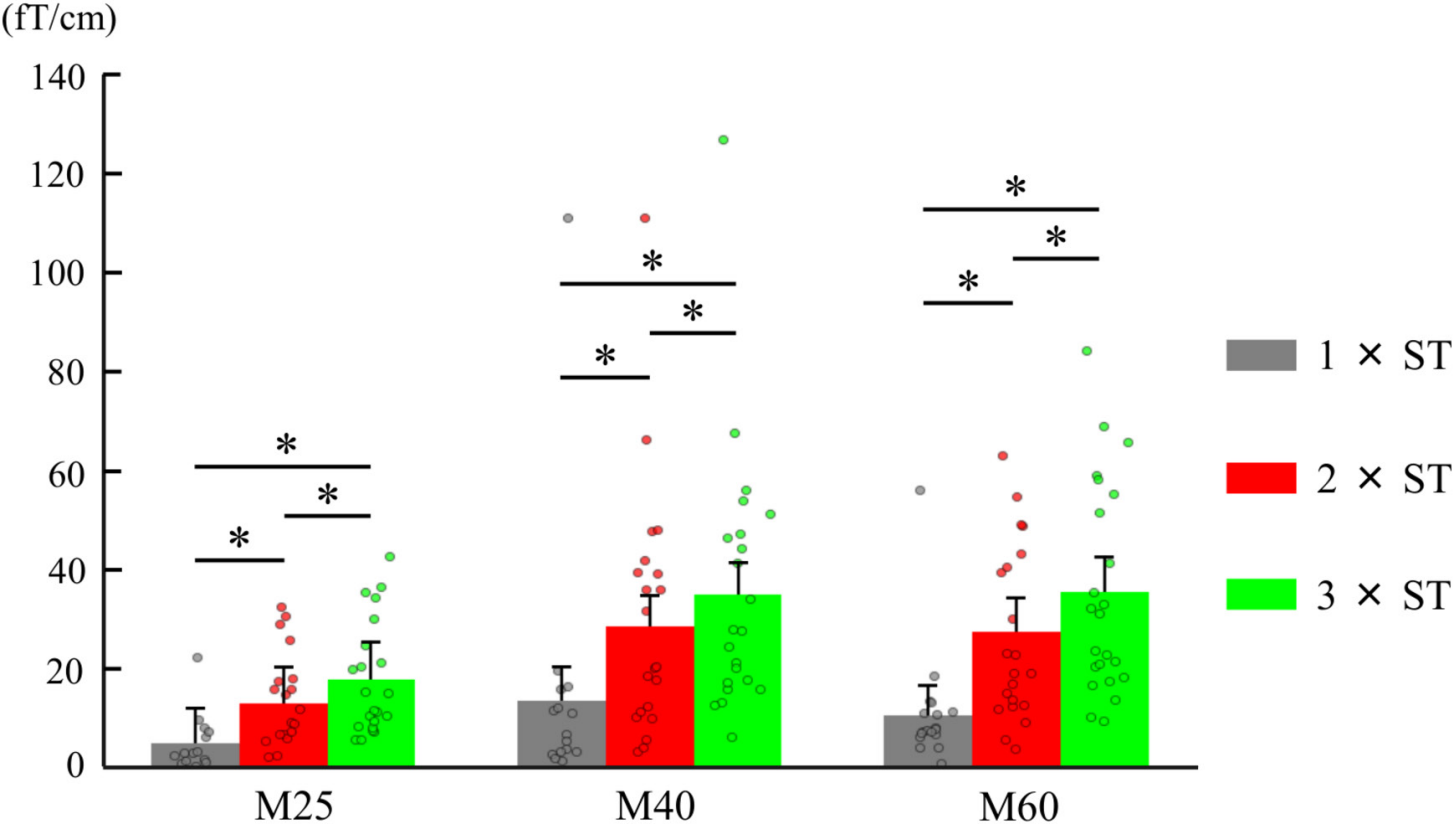
Estimated marginal mean amplitudes of SEF components (M25, M40 and M60) obtained from the linear mixed model. Error bars indicate standard errors. Black, red and green squares represent stimulation at 1×, 2× and 3 × the sensory threshold, respectively; *p < 0.01. Each dot represents an individual participant.

For each participant, the number of excluded stimulus epochs out of 300 stimuli was 29.8 ± 29.4 in 1 × ST, 31.3 ± 27.6 in 2 × ST and 30.6 ± 22.4 in 3 × ST (mean ± SD). As a result, ≥ 213 (1 × ST), 194 (2 × ST) and 215 (3 × ST) epochs were included in the averaging for each condition. Similar to βERD and βERS, SEF components were not consistently observed across all participants, particularly at lower stimulation intensities. Table 2 shows the amplitudes and latencies of the M25, M40 and M60 components and Figure 3 shows a representative RMS waveform. LMMs revealed significant main effects of stimulus intensity for all SEF components (M25, *F*_2, 18_ = 23.42, *p* < 0.01; M40, *F*_2, 18_ = 27.63, *p* < 0.01; M60, *F*_2, 21_ = 18.37, *p* < 0.01). Further, post hoc comparisons with Bonferroni correction showed significantly higher amplitudes in the 2 × ST and 3 × ST conditions than in the 1 × ST condition for all SEF components (M25, M40 and M60; all *p* < 0.01). Furthermore, the amplitudes in the 3 × ST condition were significantly higher than those in the 2 × ST condition for all components (M25, M40 and M60; all *p* < 0.01). Figure 4 shows the estimated marginal means derived from the LMMs.

**TABLE 2:** Amplitudes and latencies of SEF components.

	Amplitude (fT/cm)			Latency (ms)		
	1 × ST	2 × ST	3 × ST	1 × ST	2 × ST	3 × ST
M25	3.1 [1.8–7.4]	11.8 [6.8–17.8]	13.3 [8.7–23.9]	28 [28–32]	27 [25–28]	27 [25–28]
M40	6.7 [3.2–14.0]	20.5 [11.3–39.5]	27.9 [17.4–47.1]	43 [39–48]	41 [37–45]	40 [38–46]
M60	7.7 [6.8–11.3]	19.1 [12.6–40.7]	31.2 [19.5–53.4]	61 [57–68]	62 [55–68]	63 [57–68]

*Note:* Data are expressed as the median [IQR].

### 3.4 Performance

**FIGURE 5:**
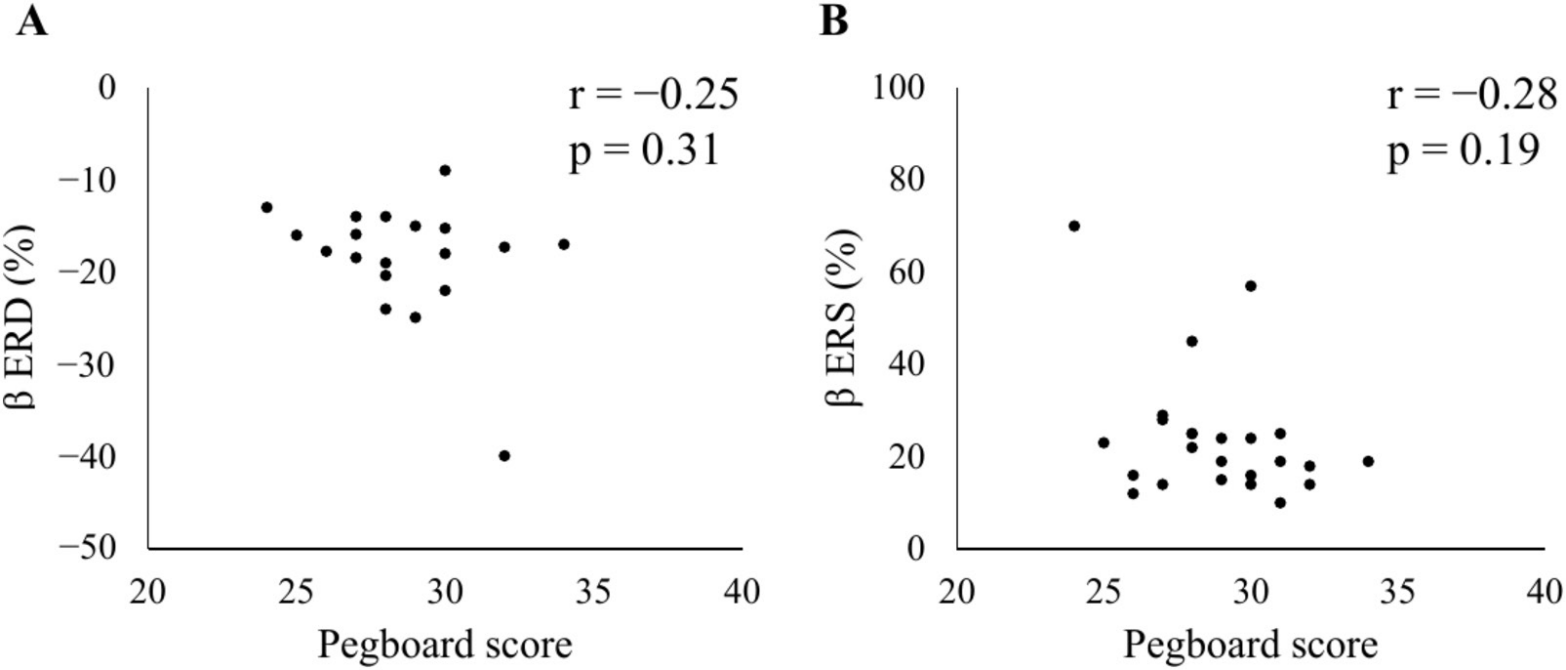
Relationship between motor performance and (A) beta event‐related desynchronization (βERD) and (B) beta event‐related synchronization (βERS). βERD data are shown for 19 participants, excluding five participants not showing βERD; βERS data are shown for all participants.

The mean pegboard score was 28.8 ± 2.4 (mean ± SD). In the 3 × ST condition, βERS was observed in all participants but βERD was not observed in five. These participants were excluded from correlation analysis. The Shapiro–Wilk test indicated that the pegboard scores followed a normal distribution (*p* = 0.968), whereas βERD and βERS amplitudes did not (βERD, *p* = 0.001; βERS, *p* < 0.001). Spearman's correlation analysis revealed that neither βERD nor βERS amplitude was significantly correlated with the pegboard score (βERD: *r* = −0.25, *p* = 0.31; βERS: *r* = −0.28, *p* = 0.19; Figure 5).

## 4 Discussion

The main findings of this study are as follows: (1) βERD and βERS amplitudes increased with the intensity of cutaneous electrical stimulation, although significant differences were only observed between the 1 × ST and 3 × ST conditions; (2) the occurrence of βERD and βERS also tended to increase with stimulus intensity; and (3) motor performance in healthy participants was not associated with the amplitude of either βERD or βERS.

### 4.1 βERD and βERS Occurrence Rates

Previous studies have not sufficiently examined the factors that modulate the occurrence rates of βERD and βERS. In the present study, these occurrence rates tended to increase with higher intensities of cutaneous electrical stimulation. These findings suggest that β‐band oscillatory modulations in response to afferent input magnitude variations are characterized not only by amplitude changes but also by the presence/absence of the response itself. In a prior study using various intensities of cutaneous stimulation (20%–80% of the pain threshold), βERS was not observed in 4 out of 13 participants at certain stimulus intensities (Stancak et al. 2003). Although specific intensity values were not reported, the absence of βERS likely occurred at lower intensities, as suggested by comparison with our findings. Similarly, in voluntary movement tasks, βERS was not observed in some participants under no‐ or low‐load conditions, whereas it appeared more consistently under higher‐load conditions (Stancak et al. 1997). Increased task load likely enhances afferent input through sensory feedback, in addition to increased efferent motor output (McCloskey 1978; Jami 1992; Boulton et al. 2021). Thus, conditions involving reduced afferent input may correspond to a higher likelihood of βERS absence.

Conversely, other studies have reported βERD in all participants, regardless of stimulus intensity or task load (Stancak et al. 1997, 2003). However, in the present study, we found that not only βERS but also βERD showed a tendency for increased occurrence with higher stimulus intensity. While previous studies defined βERD based on statistical differences from rest, we defined βERD as a decrease exceeding two SD from baseline for at least 100 ms. This methodological difference may have enabled detection of variability in βERD occurrence across different stimulus intensities. Additionally, previous research on βERD has primarily focused on motor‐related processes such as motor preparation and execution (Neuper et al. 2006; Nam et al. 2011; Peter et al. 2022). In contrast, βERD following somatosensory stimulation has been less thoroughly investigated, and it remains unclear whether its occurrence is modulated by sensory stimulation parameters. Consistent with prior findings, we confirmed that βERD can be elicited by somatosensory stimulation (Illman et al. 2020), and its occurrence rate tended to increase with stimulus intensity. These results suggest that afferent input, in addition to motor‐related processes, may influence the occurrence of βERD.

Furthermore, since many participants who did not exhibit βERD or βERS under the 2 × ST condition also failed to show responses under the 1 × ST condition, these oscillations may have threshold‐like characteristics, appearing only when the afferent input magnitude exceeds a certain level. This suggests that β‐band oscillatory modulations may not be detectable at low stimulation intensities and that a minimum level of afferent input is required to reliably record these responses after sensory stimulation. While previous studies primarily discussed the relationship between afferent input and β‐band oscillatory modulations in terms of amplitude changes, our results demonstrate that evaluating the presence or absence of a response, in addition to its amplitude, is essential for a comprehensive understanding of these responses.

### 4.2 Changes in β‐Band Oscillatory Amplitude With Increasing Stimulus Intensity

In the present study, the amplitudes of the M25, M40 and M60 components increased with stimulus intensity. The M25 component is thought to reflect excitatory postsynaptic potentials in pyramidal neurons within Brodmann area 3b, associated with afferent input, while the M40 component is considered to reflect inhibitory postsynaptic potentials in the same area or activation of area 4 (Kawamura et al. 1996; Wikstrom et al. 1996; Huttunen and Lauronen 2012; Sugawara et al. 2015; Kida et al. 2018). The M60 component has been suggested to reflect activity in Brodmann areas 1 or 2 (Huttunen et al. 2006). Torquati et al. (2002) reported that increasing the intensity of somatosensory stimulation via median nerve stimulation elicited a linear increase in activity in the primary somatosensory cortex (S1) up to a certain threshold, interpreted as recruitment of Aβ fibres until saturation. Similarly, in the present cutaneous stimulation paradigm, Aβ fibre recruitment likely scaled with stimulus intensity, leading to a greater amount of sensory information being transmitted to S1. Thus, the observed increases in M25, M40 and M60 amplitudes may reflect an intensity‐dependent enhancement of cortical afferent input. Similar to SEF components, βERS showed significantly higher amplitudes under the 3 × ST condition than under the 1 × ST condition. This observation supports previous studies indicating a strong association between afferent input and βERS (Cassim et al. 2001; Stancak et al. 2003; Houdayer et al. 2006; Illman et al. 2020).

In contrast to SEF components, βERS did not show significant differences between 1 × and 2 × ST conditions, nor between the 2 × and 3 × ST conditions. This finding suggests that βERS may not just reflect a monotonic increase in afferent input magnitude. Consistent with the present results, a previous study manipulating electrical stimulation intensity reported significantly greater βERS under strong stimulation compared to weak stimulation, and the intermediate intensity did not differ significantly from either (Stancak et al. 2003). Conversely, in voluntary movement tasks where muscle contraction force was varied, βERS amplitude exhibited a positive linear relationship with contraction strength (Fry et al. 2016). As increased muscle contraction involves heightened afferent input and increased efferent motor output (Jami 1992; Boulton et al. 2021), the βERS response during voluntary movement may differ from that elicited by the present cutaneous stimulation paradigm.

Although the physiological basis of βERS remains incompletely understood, two primary interpretations have been proposed: one posits that βERS reflects afferent input, as noted above, while the other suggests it reflects inhibitory processes within the motor cortex (Pfurtscheller et al. 1996; Stevenson et al. 2011). Supporting the latter, Chen and Hallett (1999) used transcranial magnetic stimulation over the primary motor cortex (M1) and observed reduced motor‐evoked potential amplitudes during βERS following median nerve stimulation. Moreover, Gaetz et al. (2011) reported that post‐movement βERS positively correlated with gamma‐aminobutyric acid (GABA) concentration in M1, and Pfurtscheller et al. (2005) found that βERS can also be elicited during motor imagery. Since motor imagery recruits brain regions such as the premotor cortex, supplementary motor area and M1 (Grezes and Decety 2001; Hardwick et al. 2018; Persichetti et al. 2020), βERS during voluntary movement is closely linked to motor cortical activity. Collectively, the βERS observed in the present study likely reflects predominantly afferent input and may involve different neural mechanisms from those underlying βERS during voluntary movement. Furthermore, while afferent input‐related βERS may increase in amplitude with increasing input strength, it may not be sensitive to subtle gradations in afferent input.

βERD has been extensively studied in voluntary movement tasks (Stancak and Pfurtscheller 1996; Stancak et al. 1997; Alegre et al. 2002; Kilner et al. 2005; Tzagarakis et al. 2010; Fry et al. 2016), and its amplitude has been shown to be modulated by motor preparation and planning (Kilner et al. 2005; Tzagarakis et al. 2010). Moreover, βERD appears to be independent of muscle contraction force (Fry et al. 2016), suggesting that sensory feedback accompanying voluntary movement does not substantially influence its amplitude. Conversely, the present study found that βERD amplitude was significantly greater in the 3 × ST condition than in the 1 × ST condition. This finding aligns with previous reports of enhanced βERD with increasing electrical stimulation intensity (Stancak et al. 2003). Additionally, a study comparing βERD during voluntary and passive movements showed that during voluntary movement, βERD first emerges over the contralateral central region ~1.5 s before movement onset and then appears bilaterally after movement begins (Alegre et al. 2002). Notably, bilateral βERD has also been observed during passive movement. These results suggest that βERD following movement onset may involve afferent input and differs from the βERD observed during motor preparation.

Further supporting this distinction, studies using motor imagery tasks—which lack afferent input—have reported βERD only in the contralateral central region (Pfurtscheller and Neuper 1997; Schnitzler et al. 1997). This difference in spatial distribution suggests that βERD elicited by afferent input may be underpinned by neural mechanisms distinct from those associated with voluntary movement.

### 4.3 Association With Motor Performance

In stroke patients, the amplitudes of βERD and βERS elicited by voluntary movement or somatosensory stimulation have been positively correlated with upper limb motor function at the time of assessment (Parkkonen et al. 2018; Tang et al. 2020). Conversely, the present study found no significant associations between motor performance and the amplitude of either βERD or βERS in healthy participants.

Although the precise mechanisms underlying β‐band oscillations remain unclear, they are thought to reflect the balance between excitation and inhibition within cortical networks composed of inhibitory GABAergic interneurons and excitatory glutamatergic pyramidal cells (Jensen et al. 2005; Yamawaki et al. 2008; Hall et al. 2010; Gaetz et al. 2011; Muthukumaraswamy et al. 2013). Given that stroke patients exhibit lower βERD and βERS amplitudes compared to healthy individuals (Rossiter et al. 2014; Tang et al. 2020), these oscillations may behave differently in disease‐related cortical states. Indeed, βERD and βERS amplitudes in stroke patients tend to increase in parallel with motor recovery, gradually approaching levels observed in healthy individuals (Parkkonen et al. 2018; Tang et al. 2020). Similarly, Parkinson's patients have been reported to show reduced βERD and βERS amplitudes compared to healthy controls (Pfurtscheller et al. 1998; Heida et al. 2014), further supporting the notion that β‐band oscillations are modulated by neurological conditions.

A study examining the relationship between motor learning and β‐band oscillations in healthy individuals reported that while βERD in the contralateral parietal region did not change with learning, its amplitude increased after learning (Moisello et al. 2015). As demonstrated in the present study, βERD and βERS amplitudes exhibit considerable interindividual variability (Table 1). Therefore, intraindividual changes in these amplitudes associated with motor learning may relate to motor performance, whereas interindividual differences in absolute amplitude may not directly reflect motor ability.

### 4.4 Significance of the Study

In the present study, we investigated changes in the occurrence rate of βERD and βERS in response to varying afferent input levels as well as changes in their amplitudes. Our results demonstrated that both βERD and βERS tended to increase not only in amplitude but also in their occurrence rate with increasing afferent input magnitude. This suggests that to fully understand the physiological characteristics of βERD and βERS, the presence or absence of these responses in addition to their amplitude modulations needs to be considered. Specifically, since βERD and βERS may be absent under low‐intensity stimulation, in future studies investigating β‐band oscillatory activity, stimulation intensity should be increased beyond a certain threshold to reliably induce oscillatory responses across participants.

Furthermore, βERD and βERS did not correlate with motor performance in the healthy participants included in this study. This finding supports the hypothesis that the previously reported association between beta‐band oscillations and motor function in patient populations may reflect disease‐specific alterations in cortical states rather than a universal physiological rule. Therefore, it is essential to consider the characteristics of the study population (i.e., healthy vs. clinical) when investigating and interpreting the functional significance of βERD and βERS.

### 4.5 Limitations

In the present study, stimulation intensity was limited to three levels to minimize participant burden due to discomfort and the extended duration of the experiment. Including a broader range of stimulation intensities might have enabled identification of the lower and upper thresholds required to elicit βERD and βERS as well as assessment of potential ceiling effects in their amplitudes in response to increasing stimulation intensity. Additionally, βERD and βERS may be influenced by attention and alertness (Bardouille et al. 2010; Illman et al. 2021). Although participants' alertness was monitored and verbal prompts were used to maintain wakefulness, variations in attentional engagement with the stimulation may still have affected the results.

Some participants did not exhibit detectable βERD or βERS under certain stimulation conditions. In such cases, to avoid the selection bias resulting from excluding participants with incomplete data, we treated conditions without observed responses as missing values and performed the analysis using an LMM. However, as the power analysis for this study was based on the assumption that data would be obtained across all conditions, these missing values may have decreased the statistical power.

Furthermore, there was a discrepancy between the task used to induce beta‐band oscillatory modulations and that used to evaluate motor performance. In this study, beta‐band modulations were elicited by passive cutaneous stimulation applied to a single digit, whereas motor performance was assessed using an active task requiring the coordination of multiple fingers and joints. These differences in task characteristics may have contributed to the lack of a significant correlation between the two measures. Therefore, to further elucidate the relationship between beta‐band oscillations and motor function, future research should use experimental designs with closely aligned tasks for inducing oscillatory changes and assessing motor performance. Furthermore, while selecting the frequency band showing the strongest modulation from multiple sub‐bands to account for individual variability, this data‐driven selection within the same dataset may have led to an overestimation of response amplitudes.

## 5 Conclusion

In this study, no correlation was observed between βERD and βERS induced by cutaneous stimulation and motor performance in healthy participants. Additionally, the occurrence of βERD and βERS exhibited reduced interindividual variability with increasing stimulation intensity. Although their amplitudes increased with greater sensory input, they appeared relatively insensitive to subtle variations in input magnitude. These findings may contribute to a more comprehensive physiological understanding of βERD and βERS.

## Author Contributions

**Rin Kosuge:** conceptualization, methodology, investigation, formal analysis, writing – review and editing, project administration, writing – original draft. **Mayu Akaiwa:** methodology, writing – review and editing, project administration. **Hidekazu Saito:** investigation, writing – review and editing. **Yuya Matsuda:** writing – review and editing. **Koki Iwata:** writing – review and editing. **Eriko Shibata:** writing – review and editing. **Takeshi Sasaki:** writing – review and editing, funding acquisition. **Jun Shinozaki:** resources. **Masanori Sasaki:** resources. **Yuki Ueda:** resources. **Kazuhiro Sugawara:** conceptualization, methodology, investigation, validation, supervision, writing – review and editing, project administration, funding acquisition.

## Funding

This work was supported by Japan Society for the Promotion of Science, 22K11394 and 24K14383.

## Ethics Statement

All procedures involving human participants were approved by the Research Ethics Committee of Sapporo Medical University (No. 6‐1‐26) and conducted in accordance with the 1964 Helsinki Declaration and its later amendments or comparable ethical standards.

## Consent

All participants received a detailed explanation of the experimental procedures before participation and provided written informed consent.

## Conflicts of Interest

The authors declare no conflicts of interest.

## Supporting information




**Table S1:** βERS and αERS offset time.

**Table S2:** αERD and αERS amplitudes.

**Figure S1:** (A) Time–frequency analysis (TFA) and (B) temporal spectral evolution (TSE) waveforms for a representative participant (sub22). TFA plots show the stimulation conditions at 1 × sensory threshold (1 × sensory thresholds [ST]), 2 × sensory threshold (2 × ST) and 3 × sensory threshold (3 × ST), from left to right. The two rectangles on TFA plots indicate the frequency bands and time windows used for TSE analysis of αERD and αERS, respectively. In TSE waveforms, the black line represents the 1 × ST condition, the red the 2 × ST condition, and the green the 3 × ST condition. The grey‐shaded area indicates the baseline period for analysis, whereas blue‐ and orange‐shaded areas represent the analysis windows for αERD and αERS, respectively.

**Figure S2:** Estimated marginal means of αERD and αERS amplitudes calculated using a linear mixed model. Error bars indicate the standard error. Black, red and green squares represent the stimulation conditions at 1 × sensory threshold (1 × sensory thresholds [ST]), 2 × sensory threshold (2 × ST) and 3 × sensory threshold (3 × ST), respectively; *p < 0.05. Each dot represents an individual participant.

## Data Availability

The data that support the findings of this study are available from the corresponding author upon reasonable request.
