# Supplementary material for: Cutaneous Sensory Stimulation Intensity Modulates Beta‐Band Event‐Related Desynchronization and Synchronization Amplitudes
Source: Eur J Neurosci. 2026 Jul 11;64(1):e70613. doi: 10.1111/ejn.70613 (PMC13354975; doi:10.1111/ejn.70613)
Supplement: Supplementary file 1 — Table S1: βERS and αERS offset time. [file EJN-64-0-s002.docx]

**Table S1. βERS and αERS offset time**

|  | βERS offset time (ms) | | | αERS offset time (ms) | | |
| --- | --- | --- | --- | --- | --- | --- |
|  | 1×ST | 2×ST | 3×ST | 1×ST | 2×ST | 3×ST |
| Med. | 1040 | 810 | 870 | 1130 | 1120 | 1240 |
| Max. | 1300 | 2510 | 1620 | 1580 | 1710 | 2530 |
| Min. | 790 | 630 | 610 | 690 | 640 | 710 |
