# Supplementary material for: Cutaneous Sensory Stimulation Intensity Modulates Beta‐Band Event‐Related Desynchronization and Synchronization Amplitudes
Source: Eur J Neurosci. 2026 Jul 11;64(1):e70613. doi: 10.1111/ejn.70613 (PMC13354975; doi:10.1111/ejn.70613)
Supplement: Supplementary file 2 — Table S2: αERD and αERS amplitudes. [file EJN-64-0-s004.docx]

**Table S2. αERD and αERS amplitudes**

|  | αERD amplitude (%) | | |  | αERS amplitude (%) | | |
| --- | --- | --- | --- | --- | --- | --- | --- |
| sub | 1 × ST | 2 × ST | 3 × ST |  | 1 × ST | 2 × ST | 3 × ST |
| 01 | ー | −7 | −24 |  | ー | 11 | 15 |
| 02 | ー | −8 | −11 |  | ー | 11 | 14 |
| 03 | ー | ー | −7 |  | 9 | 10 | 16 |
| 04 | −5 | −11 | −7 |  | 14 | ー | 11 |
| 05 | ー | −8 | −20 |  | 7 | 16 | 13 |
| 06 | ー | −7 | −5 |  | 5 | 6 | 23 |
| 07 | ー | −6 | −7 |  | 9 | ー | 9 |
| 08 | ー | −8 | −9 |  | 9 | ー | 12 |
| 09 | ー | ー | −12 |  | ー | 9 | 21 |
| 10 | −32 | −33 | −32 |  | 8 | 9 | 16 |
| 11 | −6 | ー | −10 |  | 5 | 33 | 57 |
| 12 | ー | −18 | −26 |  | 11 | ー | 8 |
| 13 | −6 | −7 | −11 |  | ー | 14 | 16 |
| 14 | ー | −9 | −11 |  | ー | ー | 9 |
| 15 | ー | ー | ー |  | 6 | 7 | 14 |
| 16 | −9 | −21 | −18 |  | ー | ー | 20 |
| 17 | −7 | −3 | −4 |  | ー | 7 | 11 |
| 18 | −16 | −36 | −35 |  | ー | ー | 11 |
| 19 | ー | ー | ー |  | 10 | ー | 12 |
| 20 | −10 | −40 | −44 |  | ー | ー | 7 |
| 21 | −8 | −31 | −28 |  | 8 | 11 | 13 |
| 22 | ー | −17 | −18 |  | 5 | 15 | 19 |
| 23 | ー | ー | −7 |  | 7 | ー | 14 |
| 24 | ー | −13 | ー |  | 4 | 14 | 28 |
| Median [IQR] | −8  [−5–−32] | −10  [−3–−40] | −11  [−4–−44] |  | 8  [4–14] | 13  [6–33] | 14  [7–57] |
