# Supplementary figures and images for: Cutaneous Sensory Stimulation Intensity Modulates Beta‐Band Event‐Related Desynchronization and Synchronization Amplitudes
Source: Eur J Neurosci. 2026 Jul 11;64(1):e70613. doi: 10.1111/ejn.70613 (PMC13354975; doi:10.1111/ejn.70613)

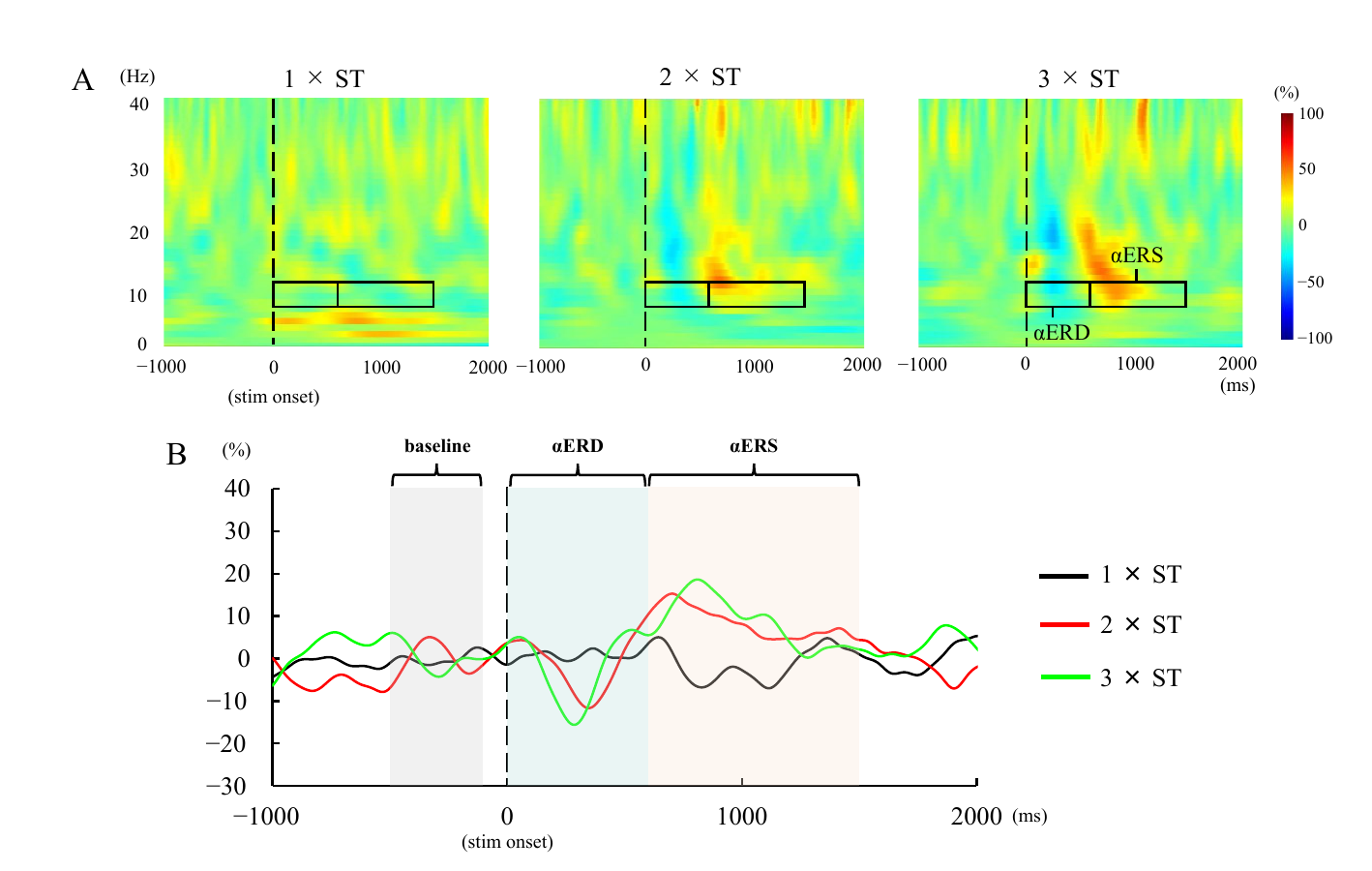

Supplement: Supplementary file 3 — Figure S1: (A) Time–frequency analysis (TFA) and (B) temporal spectral evolution (TSE) waveforms for a representative participant (sub22). TFA plots show the stimulation conditions at 1 × sensory threshold (1 × sensory thresholds [ST]), 2 × sensory threshold (2 × ST) and 3 × sensory threshold (3 × ST), from left to right. The two rectangles on TFA plots indicate the frequency bands and time windows used for TSE analysis of αERD and αERS, respectively. In TSE waveforms, the black line represents the 1 × ST condition, the red the 2 × ST condition, and the green the 3 × ST condition. The grey‐shaded area indicates the baseline period for analysis, whereas blue‐ and orange‐shaded areas represent the analysis windows for αERD and αERS, respectively. [file EJN-64-0-s003.tiff]

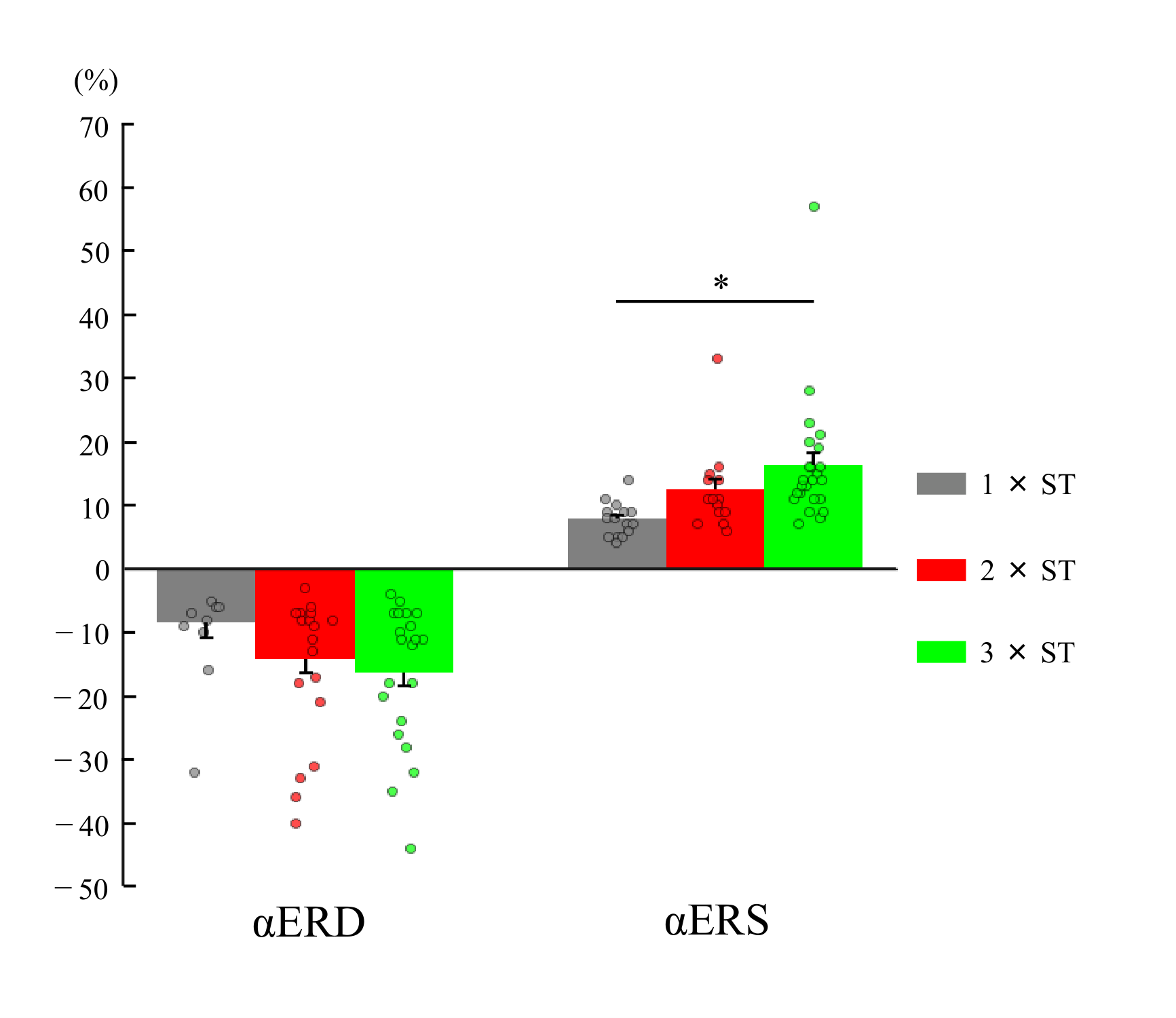

Supplement: Supplementary file 4 — Figure S2: Estimated marginal means of αERD and αERS amplitudes calculated using a linear mixed model. Error bars indicate the standard error. Black, red and green squares represent the stimulation conditions at 1 × sensory threshold (1 × sensory thresholds [ST]), 2 × sensory threshold (2 × ST) and 3 × sensory threshold (3 × ST), respectively; *p < 0.05. Each dot represents an individual participant. [file EJN-64-0-s001.tiff]
